# An Optimized Miniaturized Ultrasound Transducer for Transcranial Neuromodulation

**DOI:** 10.3389/fnins.2022.893108

**Published:** 2022-06-21

**Authors:** Chenxue Hou, Yan Wu, Chunlong Fei, Zhihai Qiu, Zhaoxi Li, Xinhao Sun, Chenxi Zheng, Yintang Yang

**Affiliations:** ^1^School of Microeletronics, Xidian University, Xi’an, China; ^2^Guangdong Institute of Intelligence Science and Technology, Zhuhai, China

**Keywords:** half-concave structure, ultrasonic transducer, converge acoustic energy, neuromodulation, FEM simulation

## Abstract

Transcranial ultrasound stimulation (TUS) is a young neuromodulation technology, which uses ultrasound to achieve non-invasive stimulation or inhibition of deep intracranial brain regions, with the advantages of non-invasive, deep penetration, and high resolution. It is widely considered to be one of the most promising techniques for probing brain function and treating brain diseases. In preclinical studies, developing miniaturized transducers to facilitate neuromodulation in freely moving small animals is critical for understanding the mechanism and exploring potential applications. In this article, a miniaturized transducer with a half-concave structure is proposed. Based on the finite element simulation models established by PZFlex software, several ultrasound transducers with different concave curvatures were designed and analyzed. Based on the simulation results, half-concave focused ultrasonic transducers with curvature radii of 5 mm and 7.5 mm were fabricated. Additionally, the emission acoustic fields of the ultrasonic transducers with different structures were characterized at their thickness resonance frequencies of 1 MHz using a multifunctional ultrasonic test platform built in the laboratory. To verify the practical ability for neuromodulation, different ultrasound transducers were used to induce muscle activity in mice. As a result, the stimulation success rates were (32 ± 10)%, (65 ± 8)%, and (84 ± 7)%, respectively, by using flat, #7, and #5 transducers, which shows the simulation and experimental results have a good agreement and that the miniaturized half-concave transducer could effectively converge the acoustic energy and achieve precise and effective ultrasonic neuromodulation.

## Introduction

Neuromodulation technology is fundamental to applications such as the probe of the nervous system and treating neurological disorders (Miniussi and Vallar, 2011; Kar and Sarkar, 2016). The available neuromodulation techniques such as deep brain stimulation, transcranial magnetic stimulation, and the newly developed optogenetics for preclinical use are either invasive or lack satisfying spatiotemporal resolution. Alternatively, transcranial ultrasound stimulation (TUS), enabling non-invasive neuromodulation with the advantages of non-invasive (Hariz, 2013; Panczykowski et al., 2014; Baek et al., 2017; Li et al., 2018), high spatial resolution, and high penetration depth, has been considered a next generation technology for probing human brain functions and treating dysfunctions (Yamaoka et al., 2011; Mead et al., 2017; Guo et al., 2021). However, since it is still in its infant stages, preclinical studies in small animals are required to figure out the fundamental mechanisms and optimal parameters for treating brain dysfunction models.

In preclinical models, it has been shown that TUS can be used to treat various brain diseases including Parkinson’s diseases, Alzheimer’s diseases, and addiction (Yang Y. et al., 2021). On the other hand, sonogenetics facilitates the non-invasive dissection of neural circuits in mouse brains. In small animal studies, anesthesia level is hard to control, which could bring additional confound to the studies as several results demonstrated that the effects of ultrasound stimulation are strongly dependent on the level of anesthesia, which can affect the reliability of experimental results (King et al., 2013; Lee et al., 2016). The use of more miniature transducers mounting on the skull is a potential solution to avoid the operation of anesthesia and immobilization. However, a small size transducer (sub-MHz) is always accompanied by low acoustic energy and spatial resolution (Zhang et al., 2020), so appropriate stimulation conditions and methods for converging acoustic energy become the critical factor of neuromodulation.

**FIGURE 1:**
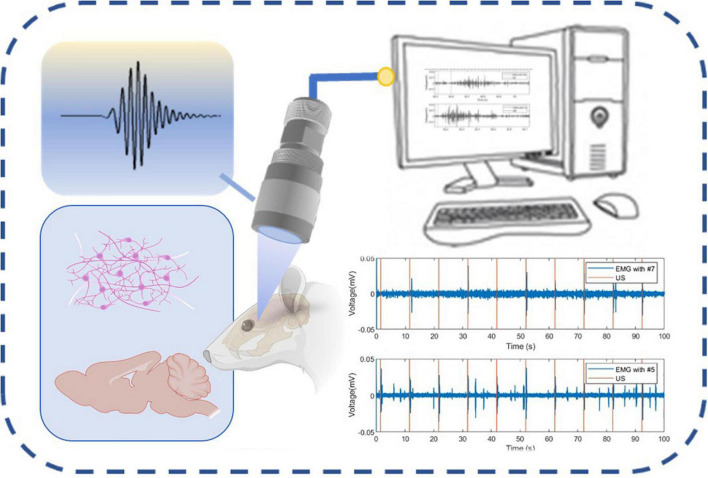
Schematic diagram of the neural regulation process.

With the development of neural regulation technology, commercial planar ultrasonic transducers gradually cannot meet the precise neural regulation. In recent years, some studies have used focused ultrasonic transducers to improve accuracy (Yoo et al., 2011; Deffieux et al., 2013; Kim et al., 2014). In this study, we proposed a transducer that both has a small wearable size and a half-concave structure to converge acoustic energy, as shown in Figure 1. The half-concave structure was made by the dimple grinder. The finite element simulation software PZFlex was used to design and analyze the single element ultrasonic transducer with different dimpling curvatures. Muscle activity in mice was induced by ultrasonic transducers with different structures. The results indicate that the half-concave ultrasonic transducer can achieve more effective convergence of sound energy and make more acoustic energy transmit through the skull when compared to the plane transducer, which leads to a higher success rate.

## Methods and Results

**FIGURE 2:**
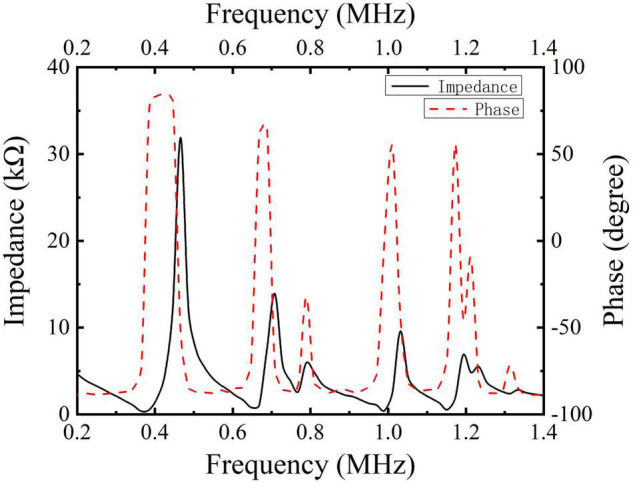
Electrical impedance and phase plots of pic255.

The characterization of material properties is the first and critical step in the design of devices because the piezoelectric dielectric properties of materials will directly affect the final performance of the device, which is why scholars have studied it extensively over the past years (Kuzenko, 2021; Yang L. et al., 2021). Several kinds of literature have pointed out that the energy transmission efficiency is higher when the ultrasonic stimulation frequency is lower than 1 MHz (Hayner and Hynynen, 2001; White et al., 2006), so it is suitable to study the acoustic characteristics of ultrasonic transducers with different structures at 400 kHz. Due to the impedance matching, the diameter of the piezoelectric element of a conventional 400 kHz transducer is much higher than 85 mm. Inversely, the diameter of the half-concave transducer is just 5 mm, and the piezoelectric element is 2 mm in thickness. Therefore, a miniaturized device is designed through half-concave structural optimization. The electrical impedance and the phase plots of the piezoelectric element were measured using the WK6500B 1J65120B impedance analyzer (Wayne Kerr Electronics, United Kingdom), as shown in Figure 2.

**FIGURE 3:**
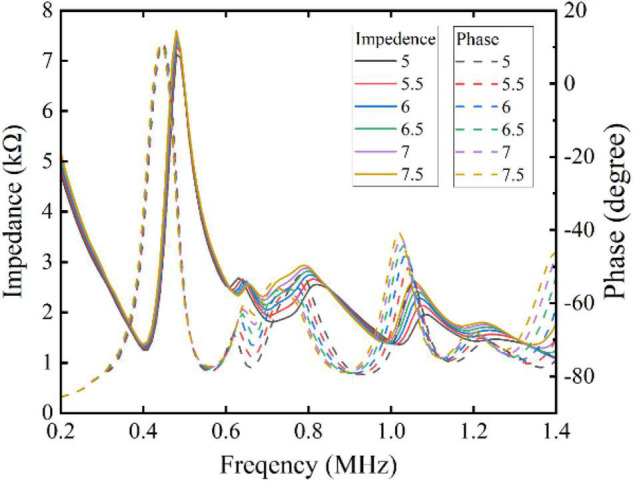
The impedances of different curvatures of piezoelectric element.

As shown in Figure 2, the measurement plot of the planar piezoelectric indicates that there are a lot of resonance peaks, and in order to further determine the excitation frequency for subsequent experiments, the spectrum of different curvatures was simulated with the help of finite element simulation. In the simulation results of the piezoelectric element in Figure 3, it is seen that there is a resonance frequency near 1 MHz, but with the change of curvature, the resonant peak has a certain deviation, but at 400 kHz, resonance coincidence is better.

The finite element simulation has been proved to be an efficient simulation method by many previous studies (Hou et al., 2021; Li et al., 2021b; Lin et al., 2021). In this study, the finite element simulation model was established to design the half-concave structure and simulate the performance of the half-concave ultrasonic transducer with the software PZFlex and Comsol. All materials used in this simulation are listed in Table 1.

**TABLE 1:** Materials used in simulation.

Function	Material	c (m/s)	(kg/m^3^)	Z (MRayl)
Piezoelectrics	Pic255	4,000	7,800	31.2
Backing layer	Epoxy	2,658	1,146	3.05
Front-load	Water	1,500	1,000	1.50

**FIGURE 4:**
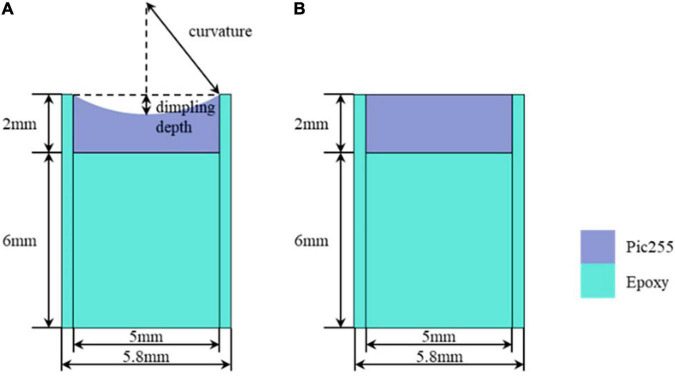
Simulation structure: **(A)** The half-concave ultrasonic transducer and **(B)**. The plane ultrasonic transducer.

The simulation parameters are as follows. The thickness of the backing material is set to 6 mm in case of backpropagation. Combined with the limitation of the actual manufacturing process, the dimpling curvature of the half-concave ultrasonic transducer was increased from 5 to 7.5 mm at equal intervals of 0.5 mm. Besides, an insulating epoxy that surrounds the piezoelectric material is 0.4 mm in width to make the condition of simulation consistent with the experiment. During the simulation, the ultrasonic transducer is excited by one cycle of sinusoidal signal with a frequency of 400 kHz and a driving voltage of 1 V peak-to-peak. Figures 4A,B show the structure diagram of the plane ultrasonic transducer and the half-concave ultrasonic transducer in simulation, respectively. The blue part represents the epoxy that acted as the backing layer and the insulating, and the purple part represents the piezoelectric material, and a reflecting plate is placed at the end of the water body, which is considered as the focal area.

**FIGURE 5:**
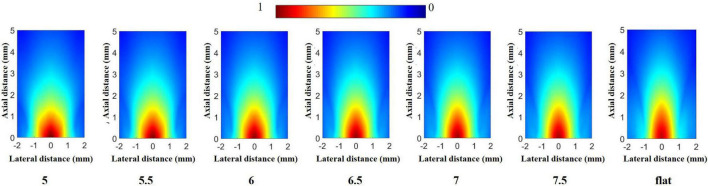
Emission acoustic field in the simulation.

Under the above parameters, a normalized emission sound field is obtained through MATLAB processing, as shown in Figure 5. Due to the excitation frequency, the wavelength of the emitted sound wave is similar to the size of the transducer, so the transducer can be regarded as a point source, and the acoustic wavefront is similar to the spherical wave (Crew et al., 1900; Simon et al., 2015). The maximum acoustic pressure data obtained from the emission acoustic field are listed in Table 2. It can be seen that the difference in the maximum acoustic pressure between the transducers with different radii of dimpling curvature is slight, but the maximum acoustic pressure of the half-concave ultrasonic transducer is slightly larger than that of the plane transducer, and the area with higher energy in the acoustic field is also larger than that of the plane transducer, which demonstrates that half-concave transducer can effectively converge the sound energy and is more beneficial to the neuromodulation of small animals.

**TABLE 2:** Maximum acoustic pressure.

Curvature (mm)	5	5.5	6	6.5	7	7.5	Flat
Pmax(pa)	3.3995e3	3.3594e3	3.3233e3	3.2936e3	3.2680e3	3.2513e3	3.1640e3

**FIGURE 6:**
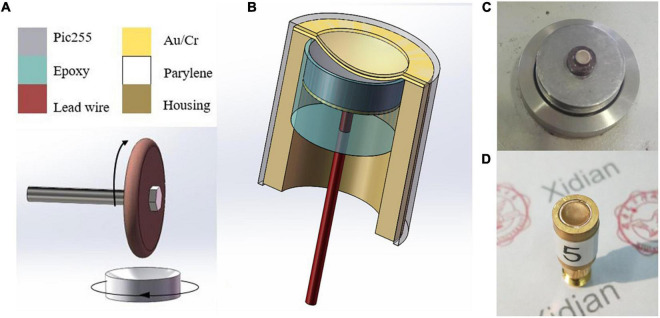
**(A)** Manufacture process of dimpling, **(B)** the structure diagram of the half-concave ultrasonic transducer, **(C)** dimpled pic255 piezoelectric ceramic, and **(D)** the packaged ultrasonic transducers.

Based on the simulation results, subsequent experiments were carried out. The manufacturing process of the half-concave structure and the structure diagram of the dimpled ultrasonic transducer are shown in Figures 6A,B. As the piezoelectric material, the piezoelectric ceramic pic255 with double-side silver electrodes was prepared in advance. Then one side of the pic255 piezoelectric ceramics was dimpled to a half-concave structure by the dimple grinder (model656, Gatan), and a 0.9 mm diameter lead was attached to the backside of the dimpled using conductive epoxy e-solder 3022 (VonRoll Isola, New Haven, CT). After that, the piezoelectric material was placed at the center of the brass housing, and then the housing was filled with insulating epoxy (EPO-TEK 301) to act as both a backing and an insulating layer. After epoxy curing, a layer of gold (∼200 μm) was sputtered on the dimpled side to act as the front electrode, and subsequently, a thin layer of parylene (∼1.5 μm) was vapor-deposited by a PDS 2010 Labcoator (Specialty Coating Systems, Indianapolis, IN) to protect the ultrasonic transducer. Finally, an SMA connector was attached to the ultrasonic transducer. The dimpled piezoelectric ceramic pic255 and the packaged ultrasonic transducer are shown in Figures 6C,D, respectively. Besides, a plane transducer was fabricated as the control group.

**FIGURE 7:**
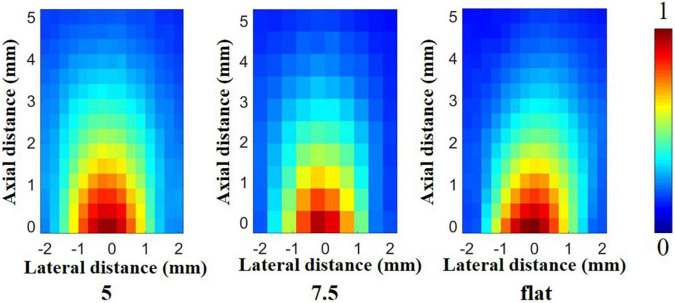
Emission acoustic field in the experiment.

The laboratory multi-function ultrasonic testing system based on LabView software was a high precision test platform (Li et al., 2021a,c). Based on the platform, the emission acoustic fields of ultrasonic transducers with different structures were measured at 400 kHz. In deionized water, the transducer was placed opposite a needle hydrophone (NH0200, Precision Acoustics, Dorchester, United Kingdom). Additionally, the acoustic pressure was measured in the range of 4 mm^2^ × 5 mm^2^ in the axial plane of the transducer. Then it was used to get the normalized emission acoustic field with MATLAB software, as shown in Figure 7. It can be seen that the emission acoustic fields of different structures of ultrasonic transducers are similar, and the difference in the maximum acoustic pressure is slight in the experiment, which is basically consistent with the simulation results.

**FIGURE 8:**
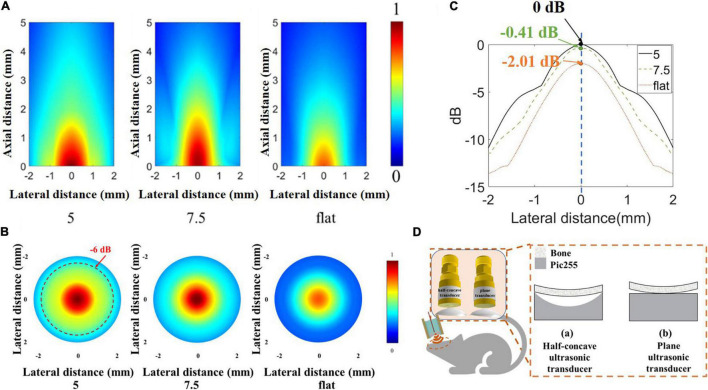
**(A)** Normalized acoustic field after transmitting through the skull by simulation. **(B)** Normalized lateral acoustic field distribution of different transducers by simulation at a maximum acoustic pressure point. **(C)** The normalized acoustic pressure distribution of the focal area in the X-Y plane. **(D)** The relative position of the skull and different transducers.

However, in biological applications, it is not comprehensive to consider only the emission acoustic field of ultrasonic transducers in water. In order to further prove the ability of the half-concave ultrasonic transducer to carry out biological experiments, a skull with a thickness of 0.5 mm was placed in front of the transducer, and the finite element simulation software PZFlex was also used to study the acoustic field distribution after transmitting through the skull. All models were set in water. The simulation model of the skull with a radius of curvature of 9 mm appressed to the surface of the transducer was established, and the relative position of the skull and the transducer is shown in Figure 8D, in which the right inset shows the skull appressed to piezoelectric material of different transducers. MATLAB software was used to process the acoustic field data after transmitting it through the skull. The normalized acoustic field was obtained as shown in Figures 8A,B. The red dash line shows the –6 dB line in the acoustic pressure distribution and the normalized lateral acoustic field distribution of different transducers at maximum acoustic pressure point is shown in Figure 8C, which shows the area with higher acoustic field energy can fall in the brain area that needs neuromodulation as far as possible. The maximum acoustic pressure is shown in Table 3.

**TABLE 3:** The maximum acoustic pressure of three transducers types.

Type	5	7.5	Flat
dBmax	0	–0.41	–2.10

It can be seen that the dimpling structure allows more energy to transmit through the skull, and the maximum acoustic pressure of the half-concave ultrasonic transducer is increased by about 20% compared with the plane transducer. The dimpling depth is greater, and the maximum acoustic pressure is greater. The greater dimpling depth achieves the greater focus effect which shows the higher resolution and near focus depth. So, the effective focus and short transmission distance can achieve higher sound energy. In summary, the half-concave structure can converge acoustic energy, and the curvature of the half-concave ultrasonic transducer is more suitable for the head of small animals. Different ultrasonic transducers were used to induce muscle activity in mice to verify the biological application value of the half-concave structure.

**FIGURE 9:**
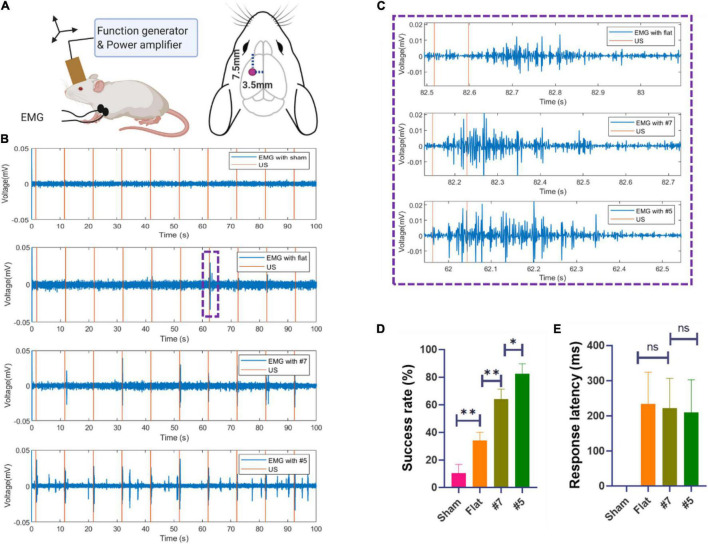
**(A)** Schematic diagram of *in vivo* mouse EMG recordings using ultrasound neuromodulation to the motor cortex. **(B)** Example sync pulses (orange) and EMG responses (blue) resulting from sonication with sham, flat transducer, focused transducer #7.5, and #5 at 1.6 MPa. **(C)** Representative EMG signals induced by a single trial, zooming from **(B)**. **(D)** The success rate of ultrasound evoked responses and the response latencies **(E)** sonicated by sham, flat, focused transducer #7.5, and #5 (*N* = 6, 20 trials/animal, *p < 0.05, **p < 0.01, one-way ANOVA with *post-hoc* Tukey test. All statistically significant differences are shown).

To further demonstrate the neuromodulation effects by using the miniaturized transducers, we stimulated the mouse brain with these transducers and monitored the motor responses determined by electromyography (EMG). The EMG recording procedures were similar to previous work (Qiu et al., 2021). Briefly, mice underwent anesthetic induction with 3% isoflurane in 100% oxygen and were maintained on 0.6% isoflurane for TUS. Eye ointments were applied to both eyes. Two 32-gauge enamel-coated copper electrodes were inserted into the triceps muscles of both forelimbs. After the attachment of all leads, the US transducer was fixed to a three-axis positioning system. Ultrasound gel was used to couple the transducer to the head of the mouse. For all experiments, the transducer was positioned approximately 1 mm from the surface of the animal’s head, targeting the primary motor cortex as shown in Figure 9A. A heat lamp distant from the experiment table was used to keep the animal warm. All the stimulation and recordings were finished within 5 min. After each mouse experiment, the EMG leads were removed, and the mouse was transferred to an induction chamber for recovery and then returned to its cage. The EMG signals were amplified with a gain of 1,000, and the bandpass was filtered between 10 Hz and 1 kHz with a preamplifier (World Precision Instruments, Sarasota, FL, United States). Data were acquired at a 2 kHz sampling rate (Lab-Jack U3, LabJack, Lakewood, CO, United States). The sonication parameters (PRF 1 kHz, 40% duty cycle, 200 ms) were controlled by a computer running software written in MATLAB (Mathworks, Natick, MA, United States). As shown in Figure 9B, The miniaturized transducers can induce EMG responses in mice experiments. The focused transducers resulted in a higher success rate compared to the flat transducer. The EMG signals were post-processed and analyzed using additional software written in MATLAB. For each trial, the DC drift was removed with a 10 Hz Butterworth IIR filter, followed by a notch filter for line frequency (60 Hz) removal. The signal was then full-wave rectified and smoothed with a 15-point moving average filter. A signal was considered representative of a muscle contraction if two conditions were met: First, the smoothed EMG signal exceeded a contraction threshold, defined as 6 SDs of the signal 100 ms before sonication plus the mean of the signal during this period. Second, the smoothed EMG signal exceeded the contraction threshold within a temporal latency of less than 200 ms. The representative responses was shown in Figure 9C. The response was then calculated for each set of ultrasound signals as a success rate defined as the number of responses divided by the total number of sonications. As shown in Figure 9D, the stimulation success rates were (32 ± 10)%, (65 ± 8)%, and (84 ± 7)%, respectively, by using flat, #7, and #5 transducers. The response latencies (Figure 9E) showed a decreasing trend, but there is no significant difference. These results were expected as these transducers have different ultrasound intensities transmitted through the skull, as shown in Figure 9.

## Conclusion

In this study, a new structure of the miniaturized ultrasonic transducer was proposed for neuromodulation, and one side of the piezoelectric material was dimpled to form the half-concave structure. Both simulated and experimental emission acoustic fields show that the half-concave structure converges the acoustic energy so that the higher energy areas of the sound field fall in brain regions where neuromodulation is needed, which would lead to more effective neuromodulation. Besides, the size of the half-concave ultrasonic transducer is smaller than most transducers in neuromodulation-related articles, which makes the biological experiment more flexible. To verify the validity of the design, different transducers were used to induce muscle activity in mice, and the result shows the half-concave ultrasonic transducer led to a higher success rate. Using the fabricated transducer with flat, #7, and #5, the stimulation success rates were (32 ± 10)%, (65 ± 8)%, and (84 ± 7)%, respectively. All results indicate the research on the half-concave structure could promote the development of neuromodulation.

## Data Availability Statement

The original contributions presented in this study are included in the article/supplementary material, further inquiries can be directed to the corresponding author/s.

## Ethics Statement

All animal experiments were approved by the Animal Subjects Ethics 7 Sub-Committee (ASESC) of the Hong Kong Polytechnic University. Animal use and 8 care were performed following the guidelines of the Department of Health—Animals 9 (Control of Experiments) of the Hong Kong SAR government.

## Author Contributions

CH, YW, and XS: drafting and refining the manuscript. ZL and CZ: conducting of the experiment and simulation. CF, ZQ, and YY: critical reading of the manuscript. All authors have read and approved the manuscript.

## Conflict of Interest

The authors declare that the research was conducted in the absence of any commercial or financial relationships that could be construed as a potential conflict of interest.

## Publisher’s Note

All claims expressed in this article are solely those of the authors and do not necessarily represent those of their affiliated organizations, or those of the publisher, the editors and the reviewers. Any product that may be evaluated in this article, or claim that may be made by its manufacturer, is not guaranteed or endorsed by the publisher.
